# Supplementary figures and images for: Label-free quantitative proteomic analyses of mouse astrocytes provides insight into the host response mechanism at different developmental stages of Toxoplasma gondii
Source: PLoS Negl Trop Dis. 2023 Sep 18;17(9):e0011102. doi: 10.1371/journal.pntd.0011102 (PMC10538781; doi:10.1371/journal.pntd.0011102)

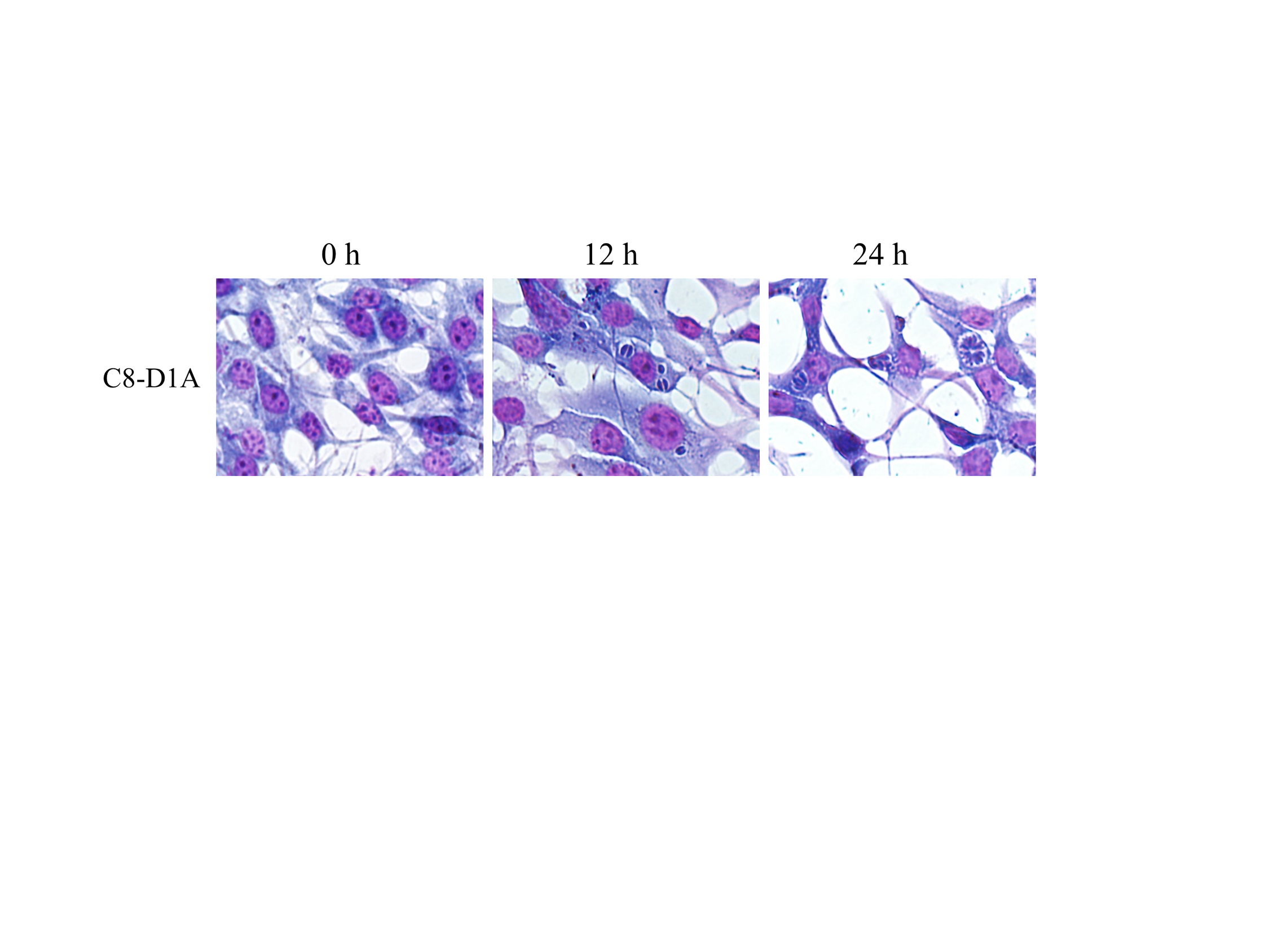

Supplement: S1 Fig — (TIF) [file pntd.0011102.s001.tif]

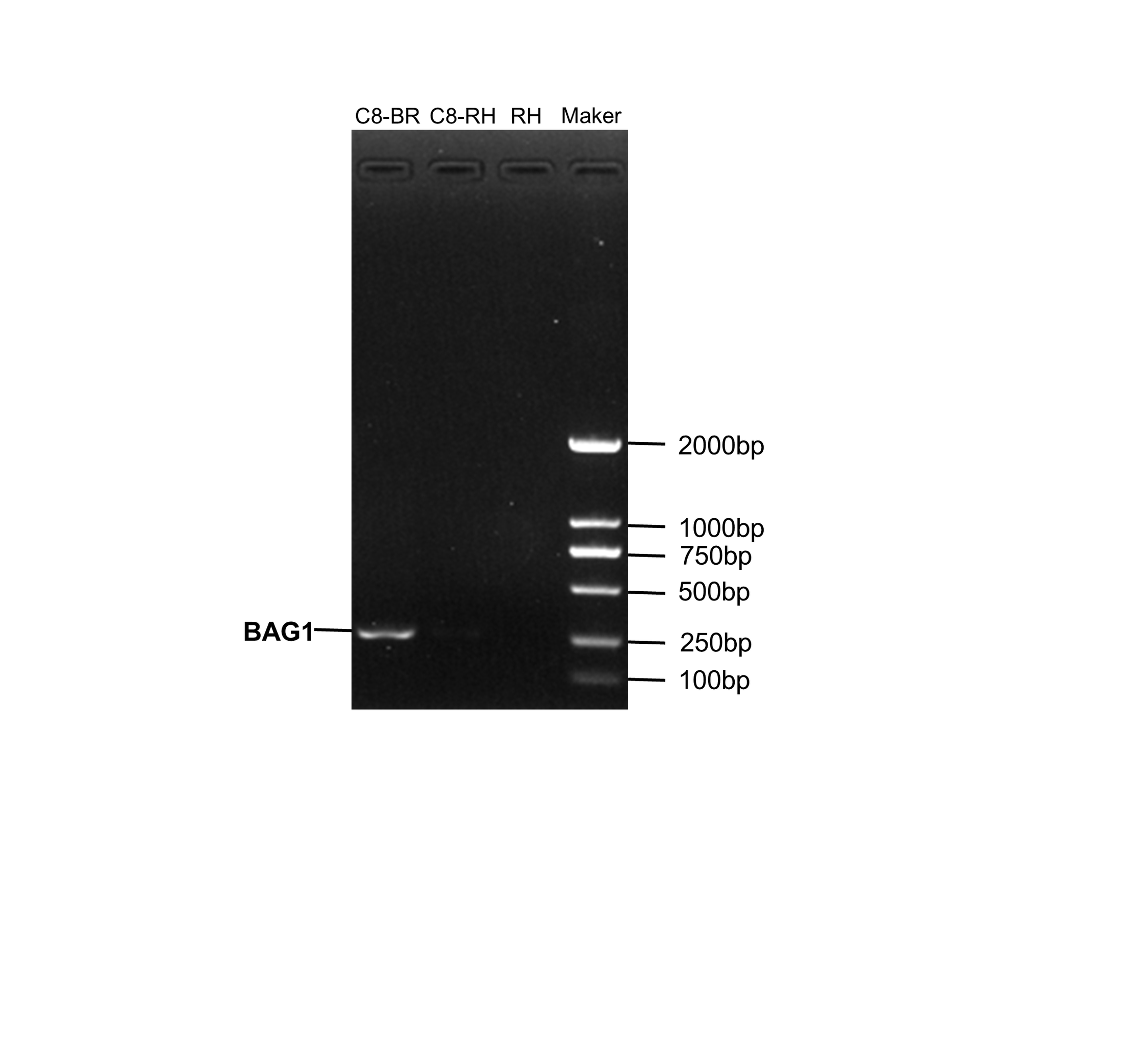

Supplement: S2 Fig — (TIF) [file pntd.0011102.s002.tif]

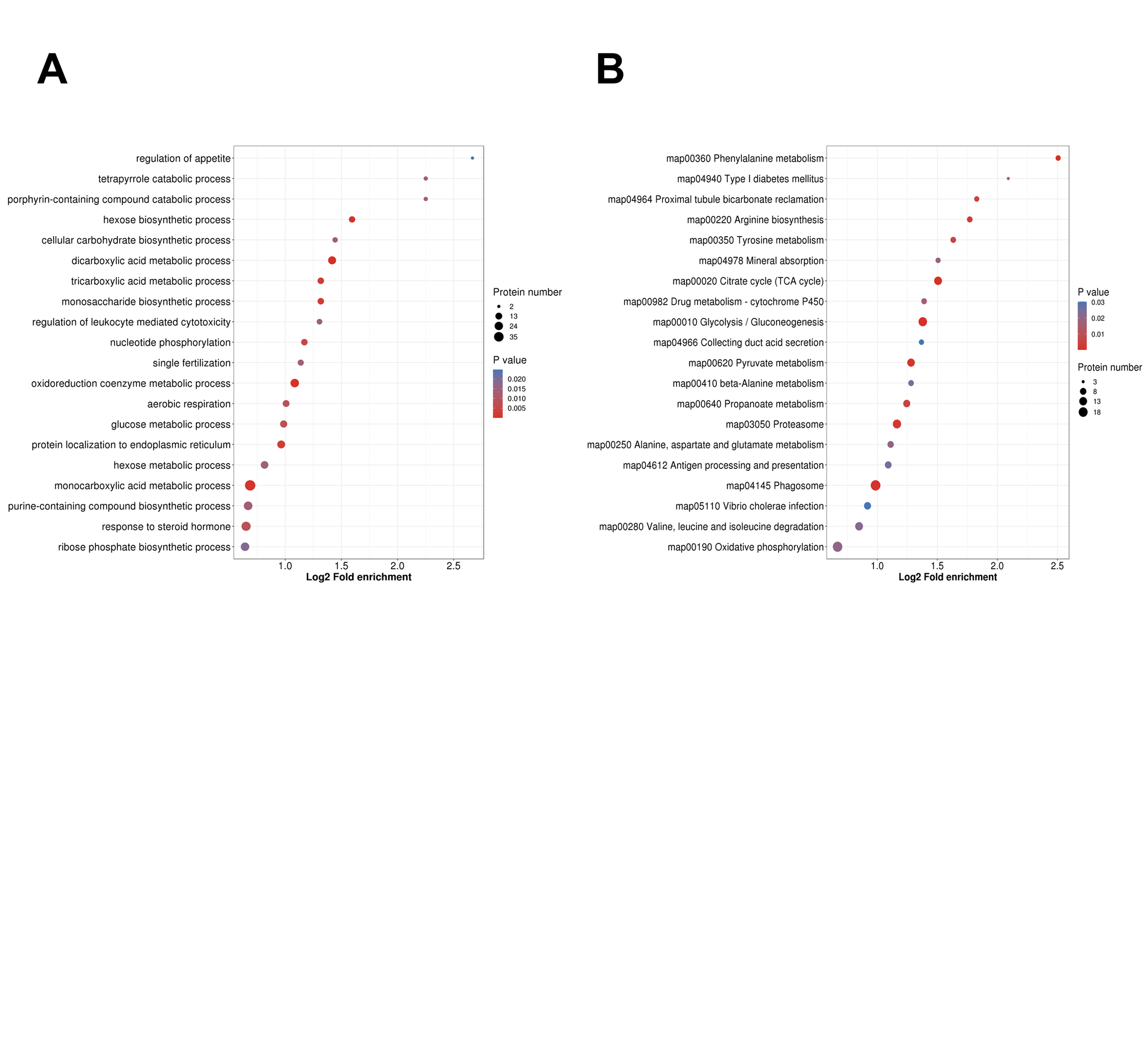

Supplement: S3 Fig — (A) GO enrichment analysis of all differentially expressed proteins. (B) KEGG enrichment analysis of all differentially expressed proteins. (TIF) [file pntd.0011102.s003.tif]
